# Establishment and molecular characterization of the novel cutaneous squamous cell carcinoma cell line from advanced-stage Indian patient

**DOI:** 10.1007/s13577-025-01237-4

**Published:** 2025-05-25

**Authors:** Darshan Mehta, Akshay Paradkar, Prakash Nayak, Bharat Rekhi, Bhabani Mohanty, Pradip Chaudhari, Sanjeev K Waghmare

**Affiliations:** 1 Stem Cell Biology Group, Waghmare Lab, Cancer Research Institute, Advanced Centre for Treatment Research and Education in Cancer (ACTREC), Tata Memorial Centre, Kharghar, Navi Mumbai, Maharashtra 410210 India; 2 Homi Bhabha National Institute, Training School Complex, Anushakti Nagar, Mumbai, 400085 India; 3 Tata Memorial Hospital, Homi Bhabha National Institute, Mumbai, India; 4 Department of Pathology, Tata Memorial Hospital, Homi Bhabha National Institute, Mumbai, India; 5 Small Animal Imaging Facility (SAIF), Advanced Centre for Treatment Research and Education in Cancer (ACTREC), Tata Memorial Centre, Kharghar, Navi Mumbai, Maharashtra 410210 India

**Keywords:** Cutaneous squamous cell carcinoma, Transmission electron microscopy (TEM), Epithelial–mesenchymal transition, In vivo tumorigenesis

## Abstract

Cutaneous squamous cell carcinoma (CSCC) is the second most prevalent skin cancer with low metastatic potential; it poses significant morbidity challenges. CSCC possesses significant heterogeneity and the treatment presents a formidable challenge. To gain a clear insight into the diverse nature of these tumors, the development of an in vitro cell line model is essential. However, there are few cell lines that were established, and only one skin SCC cell line is available on the ATCC. In the present study, we established and characterized a novel ACSCC1 cell line from the advanced-stage treatment naïve cutaneous SCC originating from the forearm of the Indian patient. The keratin expression profile showed the epithelial origin of the cell line, ploidy and karyotyping revealed the hyperdiploid population; ACSCC1 showed an increased tumorigenic and metastatic potential. Further, our cell line showed higher invasive, migratory potential and epithelial-mesenchymal-transition (EMT). Additionally, the Transmission electron microscopy (TEM) results showed an aberrant mitochondrial morphology and reduction in the cellular junctions. Further, our whole genome sequencing (WGS) analysis showed mutations in the cancer-related genes. Overall, the novel ACSCC1 cell line can be used to decipher the molecular signaling in the cancer stem cells (CSCs); targeting the CSCs population may help in understanding the tumor recurrence.

**Supplementary Information:**

The online version contains supplementary material available at 10.1007/s13577-025-01237-4.

## Introduction

Cutaneous squamous cell carcinoma (CSCC) is the second most common type of non-melanoma skin cancer after basal cell carcinoma, which arises most commonly in sun-exposed body areas due to UV radiation, chronic inflammation, chemical carcinogens, and immunodeficiency [1]. Invasive SCC is characterized histologically by the spread of malignant cells into the dermis that could arise de novo or from transforming precursor lesions such as actinic keratosis and Bowen’s disease [2]. The tumor could present clinically as a smooth or hyperkeratotic enlarging plaque, nodule, or ulcer and could be associated with pain, pruritus, or bleeding [3–6] CSCC can be categorized into primary and advanced based on the tumor size and spread. Primary CSCC is non-metastatic, while advanced CSCC is locally advanced or metastasized. Invasive SCC can recur and metastasize to regional lymph nodes or distant organs and, if left untreated or inadequately treated, can cause extensive local tissue destruction [7]. In worldwide, the incidence of CSCC has been increasing since the 1960s, which is more than 80% of non-melanoma skin cancers occur in people of 60 years old. In one cohort study of more than 1000 ethnically diverse, solid organ transplant recipients followed prospectively over 22 years, this group showed a 153-fold excess risk for developing SCC and dying as compared to the general population [8]. The 5-year survival rate of early-stage CSCC is 90%, and the treatment regime for the defined primary lesions or small tumors that are less than 1 cm is mainly surgery, excision, or cryosurgery. However, the advanced-stage cutaneous SCC is more lethal, and the five-year survival rate is 10% in case of distant metastases and 20% in case of lymph node metastasis [9, 10]. The treatment regime for advanced-stage cutaneous SCC is surgical excision and irradiation of the lymph node region after the surgery to prevent recurrence [11]. Gaining a deeper insight into cancer cells’ molecular and biological traits can contribute to developing effective strategies for preventing and identifying alternative drug targets for CSCC. The cell lines derived from primary tumors notably preserve the heterogeneous nature of the cell population observed in the original tumors. Thus, it helps to understand the detailed molecular mechanism in the CSCC. There are only a handful of well-established CSCC cell lines accessible across the globe. The online database of the ATCC, DSMZ, and ECACC revealed only a few cutaneous SCC cell lines. The commercially available cutaneous SCC cell line is A431, derived from the vulva. However, there is no evidence of UV-induced mutation and human papillomavirus (HPV) association in the A431 cell line [12–14]. Further, in 1980, Rheinwald and Beckett established two cutaneous SCC cell lines (SCC12 and SCC13B) [15]. However, the tumor characteristics and clinical details of the tumor site are unavailable, although both the cutaneous SCC cell lines are widely used for the experiments [16]. There have been reports of non-metastatic cutaneous skin SCC cell lines (UT-SCC-12 A, UT-SCC-91, UT-SCC-105, UT-SCC-111, UT-SCC-118) and metastatic cutaneous SCC cell lines UT-SCC-7, 59 A, 115 [17, 18]. However, there is a lack of evidence about the characterization of all the cell lines. All three cell lines have been used extensively for drug screening. Recently, metastatic cell line HCB-541 was established from Brazilian patient [19]. This cell line carries the TP53, HRAS, and TERT mutations and possesses high tumorigenic potential. Previously, we established the advanced stage treatment naïve three buccal mucosa (HNSCC) cell lines [20]. Importantly, there have not been any reports of the establishment of cutaneous SCC cell lines from the Indian patients. In the present study, we established advanced-stage treatment naïve cutaneous squamous cell carcinoma cell line ACSCC1 from the forearm. We confirmed the epithelial origin of the cells by performing the immunofluorescence assay (IFA) cytokeratin markers (Keratins 14 and 8). The novelty of the established cell line was determined by performing the short tandem repeat (STR) profile. Further, ploidy analysis and karyotyping were performed. The invasion and migration potential of the cells were determined by in vivo and in vitro experiments. In vivo tumorigenesis and metastasis assay showed that the cell lines possess aggressive tumorigenic and metastatic potential. Importantly, WGS analysis showed an increased number of missense mutations and C>T transitions in the whole genome of the ACSCC1 cell line. The established cell line will help to understand the molecular signaling involved in the cutaneous SCC and it can be used to screen therapeutic drugs.

## Materials and methods

### Ex-plant culture and primary cell culture

The advanced-stage (T4N0) cutaneous SCC samples were collected from the ACTREC-TMC (Navi Mumbai, India), and Tata Memorial Hospital (TMH, Mumbai, India) with patients ranging from 30 to 80 years. The patients who tested positive with HIV, HPV, and HBV were excluded from the study. The tumor biopsies were transported in sterile vials with a tissue storage solution on ice. Further, the tumor was surface-sterilized using 10% Povidone–iodine for 1 min, followed by three 1× PBS washes for 5 min each. The tumor was minced with a sterile blade and transferred to 35mm tissue culture plates with minimal essential medium (MEM) containing 10% fetal bovine serum (FBS), 10% horse serum, and 1% antibiotics. The ex-plants were maintained in the incubator at 37°C, 5% CO_2_. Further, the keratinocytes were trypsinized using 0.25% trypsin-EDTA from the ex-plant. We have stored all the passage numbers of cells in liquid nitrogen. We have used the primary human epithelial keratinocytes, adult (HEKa) (Invitrogen), as control cells. HEKa were maintained in the DMEM medium containing 10% FBS and 1% antibiotics.

### Identification of doubling time

The cells were seeded in the 60mm culture plates and maintained in the incubator at 37°C, 5% CO_2_. The cells were counted after every 24 h till 96 h. We used T.ln2/ln (Xe/Xb) to calculate the doubling time of the cells: T=Incubation time, Xe=Cells at the end of incubation, and Xb=Cells at the beginning of incubation.

### Immunofluorescence assay (IFA)

The ACSCC1 cells were fixed in the 4% paraformaldehyde (PFA) for 10 min at room temperature (RT), followed by the two 1x PBS washes at RT for 5 min The cell permeabilization was performed using the 0.1% or 0.2% Triton-X treatment for 10–15 min at RT. Further, the cells were blocked by using the 5% normal goat serum (NGS) for 1 h at RT. The primary antibodies were incubated overnight at 4°C. Next day, the cells were washed with the 1x PBS 3 times for 5 min each and incubated with secondary antibodies for 1 h at RT. Further, Hoechst (1 mM) was used for the nuclei staining for 5 min and the slides were mounted by Vectashield antifade. The images were acquired by using the Zeiss LSM780 confocal microscope. The antibody details are in Supp. Table 2.

### Novelty of the cell line by STR profiling

The STR profiles of the cell line and the original patient tumor from which the cell line was established were identified by using 16 specific STR markers. The DNA was isolated with the Genomic DNA Miniprep Kit (Qiagen, Germany). We have disclosed only eight of the STR markers to maintain patient confidentiality. The STR analysis was performed using the PowerPlex® 16 HS System and the profiles were cross-referenced with the DSMZ STR profiles database.

### HPV detection by nested PCR

We have performed Nested PCR to detect HPV in the cell line. The cell line DNA was isolated by the manufacturer’s instruction (Qiagen, Germany). Further, we have used 2 sets of primers (MY09, MY11, and Gp5+, Gp6+) to detect the HPV in the cell line. Initially, the HPV genome (450bp) was amplified using the MY09 and MY11 primers. Further, the PCR product was used to amplify the L1 region (150bp) of the virus by using Gp5+/Gp6+ primers [21].

### Identification of ploidy

The cells were collected after trypsinization and suspended in 1x PBS containing propidium iodide (0.08mg/ml). The suspension was incubated at 37°C for 30 min to stain the DNA. The DNA content of the cell line was compared with PBMCs (Diploid genomic content). Further, the fluorescence of the cells was analyzed by using an Attune Nxt FACS Calibur instrument (Thermofisher, USA). The data was analyzed using ModFit 2.0 software developed by Verity Software House, Inc.

### Determination of karyotyping

The chromosome analysis was conducted using a modified version of the conventional techniques used for karyotyping. The metaphases tended to spread excessively and the duration of trypsinization was decreased for G-banding. To determine ploidy in the cell line, we examined 50 different metaphase spreads.^20^

### Invasion assay and migration assay

We performed the invasion and migration assays as described previously [22]. We have seeded the 50,000 cells in 100µl incomplete MEM medium in the top compartment of cell culture inserts for migration assay, while 600µl incomplete MEM medium in the bottom compartment for the control group and complete MEM medium for the experimental group. Further, we have seeded 50,000 cells in an incomplete MEM medium in the upper compartment of inserts coated with 100µl Matrigel, while 600µl complete MEM medium in the lower compartment as a chemoattractant for invasion assay. The cells on the upper side of the inserts were removed with cotton swabs after 48 h. The membranes were cut from the inserts and stained with a 2% crystal violet solution. The images were taken on the Leica microscope (Leica, Germany).

### Transmission Electron Microscopy (TEM) analysis

We performed the TEM as described previously [22]. The cells were grown until they reached 90% confluency in a 35-mm culture plate. In monolayer studies, the cells were rinsed with sterile 1x PBS and treated with 3% glutaraldehyde in 0.1M sodium cacodylate-HCL, pH 7.4 for 2 h at 40 °C, and then with 1% osmium tetroxide for 1 h at 40 °C. Further, the sections were stained with lead citrate. The images were captured using TEM (Jeol 1400plus, Japan) at 120 KV. The analysis was done using the ImageJ 1.45S.

### In vivo tumorigenesis assay and tail vein metastasis assay

To assess the tumorigenic potential of the cell line, we injected one million (1 x 10^6^) cells mixed with the matrigel (3:1) subcutaneously in the flank of the NOD/SCID mice. The tumor volumes were measured by using Vernier caliper every week. Further, to understand the metastatic potential of the cell line, we have resuspended the 0.5 million (0.5 x 10^6^) cells in incomplete MEM media and injected the cells through the tail vein in the NOD/SCID mice. Further, we monitored the injected mice for 30 days. We sacrificed mice and harvested their lungs for analysis to identify metastasis by H&E staining. Moreover, we have also performed the PET-CT analysis as described previously [23] to understand lung metastasis.

### Spheroid formation assay

We have used 5000 and 10,000 unsorted cells of the ACSCC1 cell line to check the spheroid forming capacity. The cells were resuspended in the mammocult (serum-free) medium, seeded in the ultra-low attachment plates, and incubated at 37°C for 8–10 days. The images of the spheroids were taken on the Leica microscope (Leica, Germany).

### Real-Time PCR analysis

RNA extraction from cultured cells was performed using TRIZOL. The first strand of cDNA was generated using the Primescript RT reagent kit in 20ul of the final volume. The Real-Time PCR was performed on Quant Studio V (Applied Biosystems, USA) with 5ng of cDNA as a template and TB green premix. The GAPDH gene was used as an internal control for normalization. The fold change for each gene was computed using the conventional 2^-ΔΔCt technique. The primer details are mentioned in Supp. Table 1.

### Whole genome library preparation, sequencing, and analysis

Whole genome libraries were prepared at a length of 300bp using 1µg total extracted DNA. The library preparation was carried out using the Kapa Hyperplus Kit. Further, the paired-end (150bp X2) WGS was carried out at a depth of 30X using the Illumina NovaSeq X Plus platform. The quality of generated reads was tested using FastQC version 0.11.9 [24]. The raw reads were trimmed using trimmomatic version 0.39 [25]. The trimmed reads were aligned to the reference human genome (GRCh38) from Ensembl using Bwa-mem2 version 2.2 [26]. Subsequently, the marking of duplicates (MarkDuplicatesSpark) and base quality score recalibration (ApplyBQSR) were carried out to produce analysis-ready reads. Further, we carried out somatic variant calling (Mutect2), read orientation model preparation (LearnReadOrientation), calculation of contamination (CalculateContamination), filtering of called variants (FilterMutectCalls), and Annotation of variants (Funcotator). The variant calling was performed using the genome analysis toolkit (GATK4) version 4.6.1.0 [27]. The assignment of variant effect was carried out using ensemble variant effect predictor (VEP) version 113-1 [28]. The VEP-annotated VCF files were converted to MAF format using vcf2 maf version 1.6.22. The summary of the output MAF file was plotted using the Maftools R package version 2.22.0 [29].

### Statistical analysis

We performed the two-tailed t-test to analyze the statistical significance of the invasion, migration, spheroid formation assay, Real-Time PCR, and in vivo tumorigenic potential. We have used the ImageJ 1.45S software to analyze TEM images and intracellular space between the cells and a one-sample t-test to analyze the data. We used the GraphPad Prism 8 for the analysis.

## Results

### Establishment and characterization of cutaneous SCC cell line

**Fig. 1:**
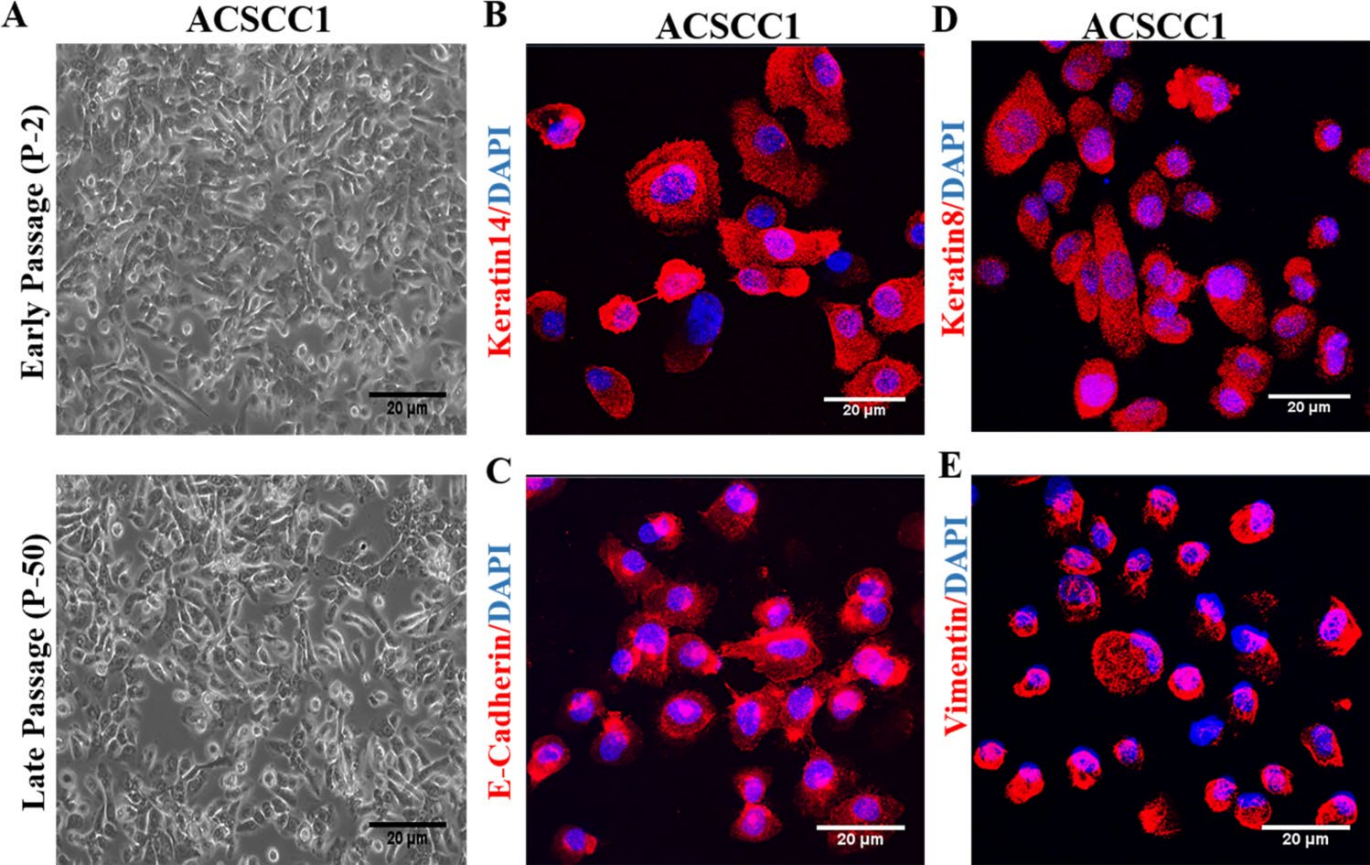
Establishment and functional characterization of cell line. **A** Representative image of the early passage of ACSCC1 cells (P-2) and late passage ACSCC1 cells (P-50). **B** Immunofluorescence staining of Keratin14 in ACSCC1 cell line n=3, independent replicates (P-7, P-8, P-9). **C** Immunofluorescence staining of the epithelial marker E-cadherin in ACSCC1 cell line n=3, independent replicates (P-7, P-8, P-9). **D** Immunofluorescence staining of the basal epithelial and transformed keratinocytes marker Keratin8 in the ACSCC1 cell line n=3, independent replicates (P-7, P-8 P-9). **E** Immunofluorescence staining of the mesenchymal marker Vimentin in ACSCC1 cell line n=3, independent replicates (P-7, P-8, P-9)

We collected advanced-stage treatment-naïve (T4N0) cutaneous SCC biopsies in the tissue storage solution processed as described previously [20]. Further, ex-plants were incubated in a complete MEM medium with medium changes every alternate day. After 1–2 weeks, compact clustered colonies were observed around the ex-plant. However, we did not observe the growth of any fibroblast in the ex-plant culture. We subcultured the keratinocytes for over 50 passages and monitored the epithelial morphology of cells **(**Fig. 1A**)**. The ACSCC1 cell line had a doubling time of 26.80 h, indicating stable morphology and long-term expansion of primary keratinocytes. Further, we observed the epithelial distinct phenotype of the ACSCC1 cell line. We checked the expression of two cytokeratin markers, Keratin 14 and Keratin 8, to confirm the epithelial origin of the cell line. IFA analysis showed a high expression of Keratin 14 (Fig. 1B) and Keratin 8 (Fig. 1D) in all the keratinocytes, suggesting that keratinocytes originate from basal epithelia and are transformed (Fig. 1B, D). Further, we checked the expression of EMT markers in the ACSCC1 cell line. Our data demonstrated the higher expression of Vimentin (Fig. 1E) and E-cadherin (Fig. 1C). The presence of Keratin 14 and Vimentin in the keratinocytes indicates a partial EMT state in the cell line. Therefore, the established cell line is highly aggressive in nature.

**Fig. 2:**
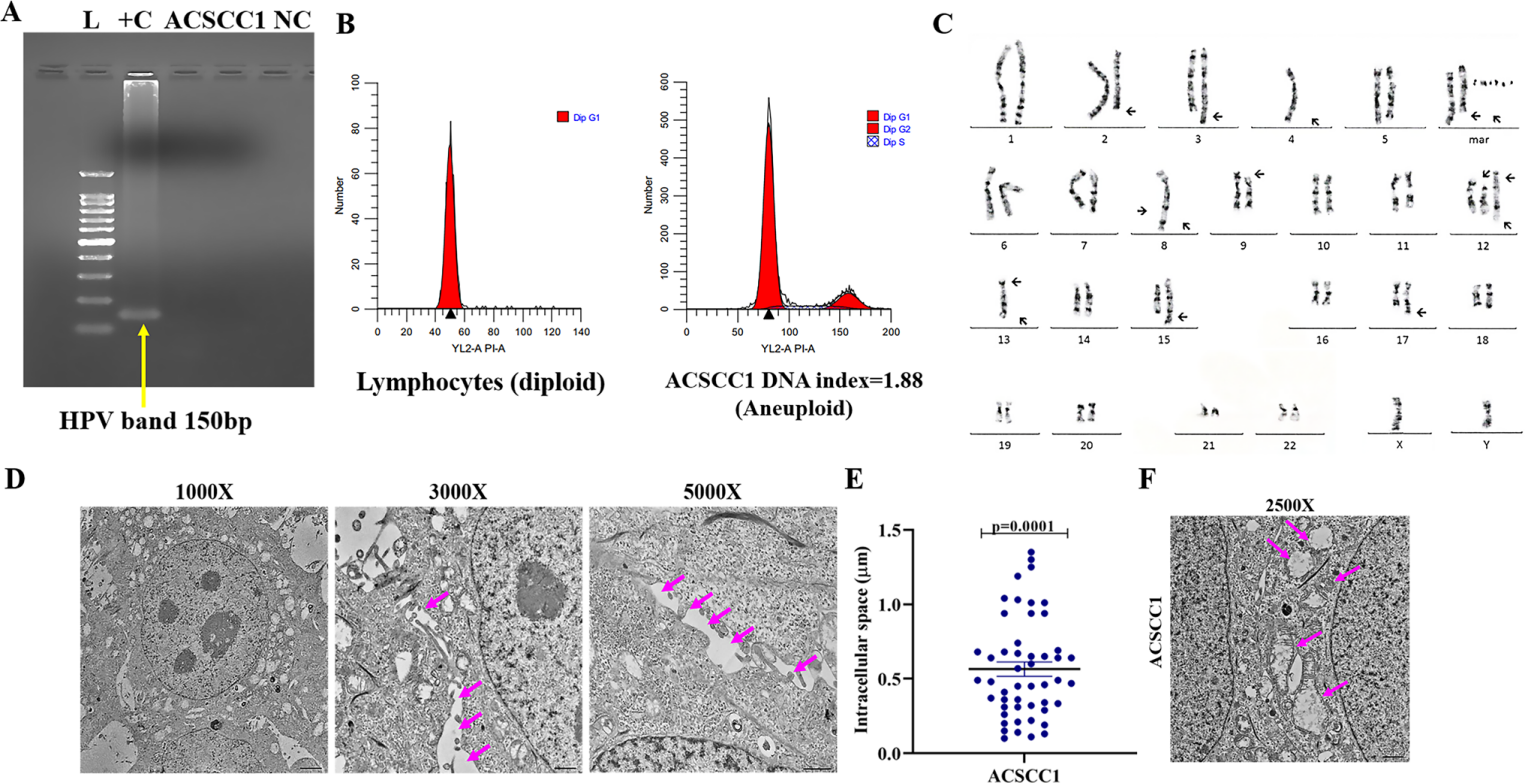
Karyotyping, ploidy analysis, and TEM analysis of cell line. **A** HPV typing of ACSCC1 cell line by using Nested PCR primers (My09/My11) and (Gp5+/Gp6+). L= Molecular ladder (100bp), +C= HeLa DNA positive for HPV band expected at 150 bp, ACSCC1 cells -Ve for HPV and NC= Negative control n=3, independent replicates (P-8, P-10, P-12). **B** Flow cytometry was used to evaluate the genomic DNA content in the ACSCC1 cell line. Normal human lymphocytes act as a control representing diploid DNA content, while the ACSCC1 cell line exhibits aneuploid DNA content n=3, independent replicates (P-8, P-10, P-12). **C** Karyotyping of the ACSCC1 cell line confirmed abnormal DNA content, with hyperdiploidy (49–52 chromosomes) and no normal metaphases. Clonal abnormalities included gains on chromosomes 3 and 12, losses on 4, 8, 13, and 15, as well as structural anomalies on 8q, 12p, 13p, 15q, 17q, and isochromosomes on Xp and 3. Deletions were observed on 2q, 9p, and 12p. A total of 14 metaphases were analyzed (P-12). **D** TEM of the ACSCC1 cell line revealed intercellular spaces within confluent monolayers, highlighting the structural features of cell-to-cell junctions and the spatial arrangement of adjacent cells (P-8). **E** Graphical representation of intracellular space between the cells by TEM. The ACSCC1 cell line showed increased intracellular length mean ± SEM, one sample *t*-test **F** TEM of the ACSCC1 cell line exhibited aberrant mitochondrial morphology within the confluent monolayers (P-8)

Recently, it has been observed that HPV may act as a co-carcinogen in cutaneous SCC patients. It attributed a role in the UV-exposed cancer type. Therefore, we performed the nested PCR to determine the HPV status in the cell line. We used the DNA of the HeLa cell line as a positive control for HPV detection. Our data showed the absence of HPV in the cell line (Fig. 2A). Further, the STR profiles of the ACSCC1 cell line were different, indicating their identity and the absence of cross-contamination of any type of cells. Moreover, the STR profile of the ACSCC1 cell line and the original patient tumor are similar, suggesting that the established cell line was derived from the advanced stage treatment-naïve (T4N0) skin SCC patient (Supp. Table 3).

### Determination of ploidy analysis and karyotyping

To understand the ploidy of the cells, we checked the DNA content of the ACSCC1 cell line as compared to the human lymphocytes, which possess the diploid content. Aberrant DNA content and amplification of the DNA indicate the hallmark of the cancer. We calculated the DNA content by dividing the mean channel of the cells in the G0 phase of the diploid lymphocytes. The DNA index of the ACSCC1 cell line showed 1.88, which is higher than the diploid DNA content (Fig. 2B). The DNA content of the ACSCC1 cell line is abnormal. Further, to confirm the results obtained from ploidy analysis, we performed the karyotyping analysis of the ACSCC1 cell line. The ACSCC1 cell line did not show any normal metaphase. ACSCC1 cell line showed the aneuploidy, with the hyperdiploidy (49–52 chromosomes). Clonal structure anomalies include additional material on the 8q, 12p, 13p, 15q, and 17q and isochromosomes, mainly Xp and 3. Further, the deletions of 2q, 9p, and 12p were observed. Loss of chromosomes 4, 8, 13, and 15 while gain of chromosomes 3 and 12 were observed. A total of 14 metaphases were karyotyped (Fig. 2C).

### Enhanced tumorigenic and metastatic potential of cutaneous SCC cell line

**Fig. 3:**
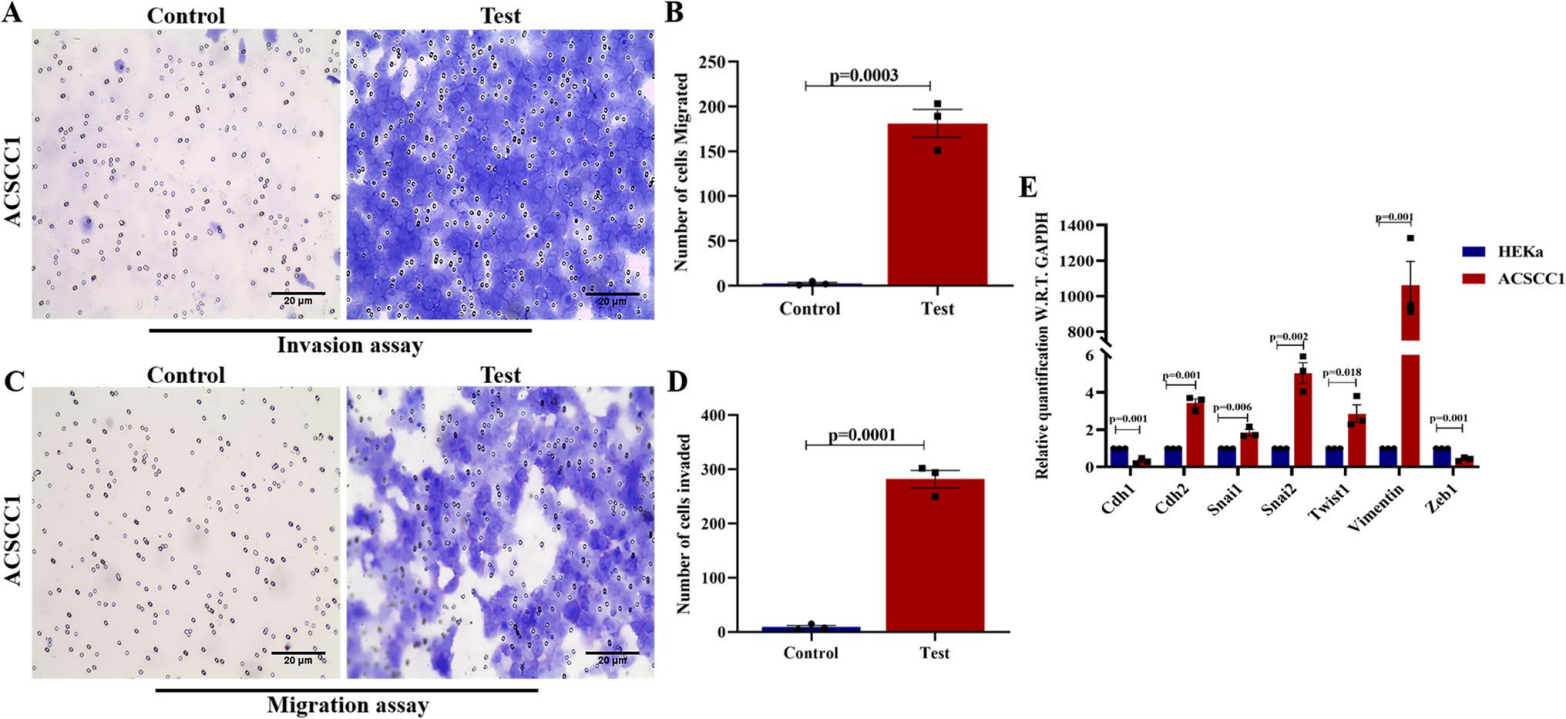
Invasion and migration potential of ACSCC1 cell line. **A** Trans-well invasion assay of ACSCC1 cells showed an increased invasive capacity, n=3, independent replicates (P-10, P-12, P-14). **B** Graphical representation of the invaded cells of ACSCC1 cell line, mean ± SEM, two-tailed *t*-test n=3, independent replicates. **C** Trans-well migration assay of the ACSCC1 cell line showed the higher migratory potential, n=3, independent replicates (P-10, P-12, P-14). **D** Graphical representation of the migratory cells of ACSCC1 cell line, mean ± SEM, two-tailed *t*-test n=3, independent replicates **E **Gene expression analysis of EMT markers *Vimentin*, *Twist1*,* Zeb1*, and *cdh1* by - Real-Time PCR, n=3, independent replicates, mean ± SEM, two-tailed *t*-test (P-10, P-12, P-14).

**Fig. 4:**
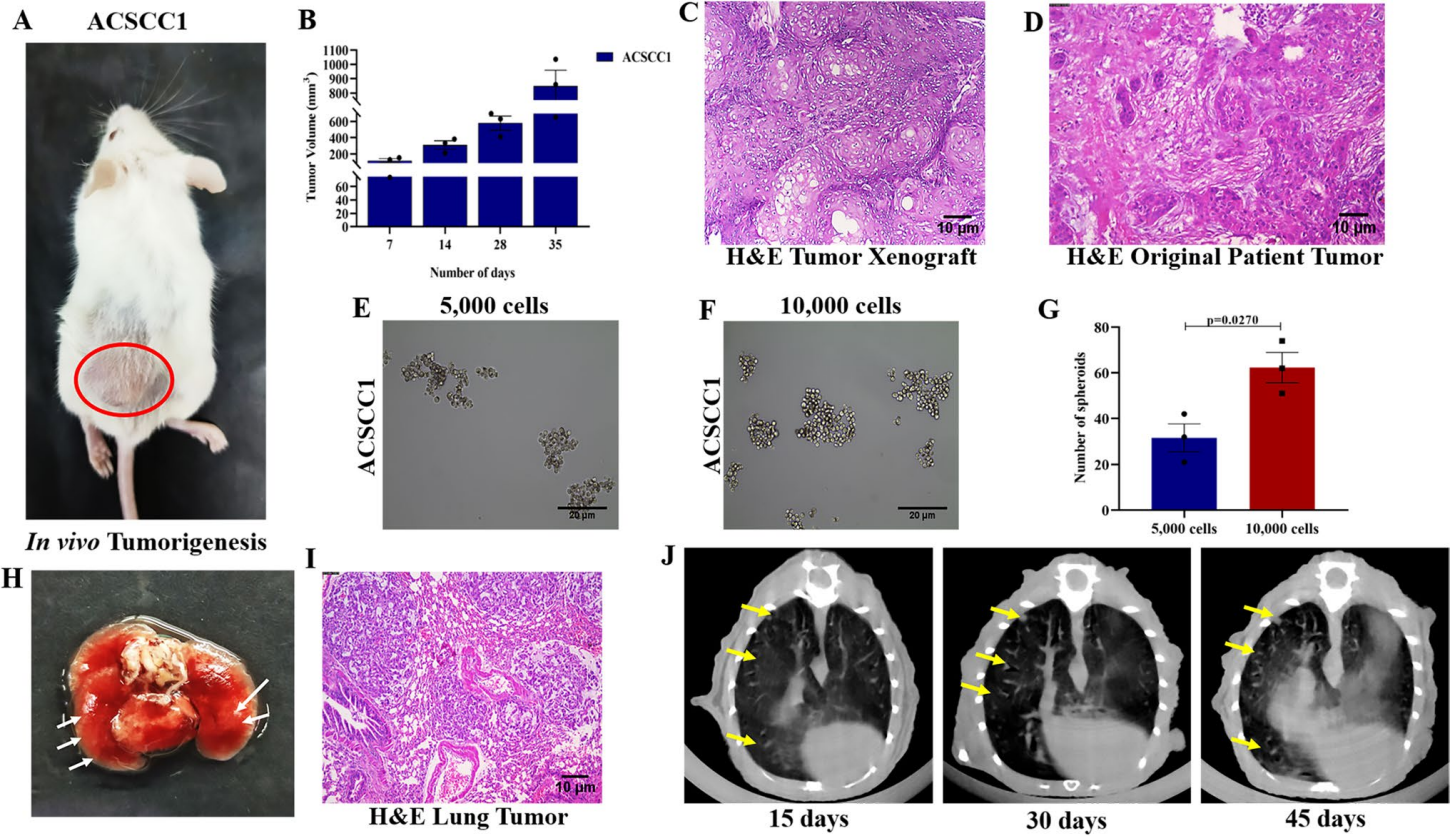
In vivo tumorigenic potential of ACSCC1 cell line. **A** In vivo tumorigenesis of the ACSCC1 cell line by injecting the one million cells subcutaneously in the flank of NOD/SCID mice, n=3, independent replicates (P-10, P-12, P-14). **B** Graphical representation of the tumor volume of ACSCC1 in vivo tumorigenesis assay, mean ± SEM, two-tailed *t*-test n=3, independent replicates. **C** Histological analysis of the ACSCC1 xenograft tumor by H&E staining showing the poorly differentiated squamous cell carcinoma, *n*=3, independent replicates (P-10, P-12, P-14). **D** Histological analysis of the original patient tumor from which cell line was established by H&E staining showed the poorly differentiated squamous cell carcinoma. **E** & **F**) Spheroid formation assay of 5000 and 10,000 ACSCC1 cells seeded in the anchorage-independent ultra-low attachment plate n=3, independent replicates (P-13, P-14, P-15). **G** Graphical representation of the number of spheroids formed by the ACSCC1 cell line, mean ± SEM, two-tailed *t*-test n=3, independent replicates. **H** Representative image of lungs showing the in vivo metastatic colonies n=3, independent replicates (P-13, P-14, P-15). **I** Histological analysis of the lung tumor by H&E staining. **J** PET-CT analysis of the in vivo lung metastasis showed an increased metastatic potential of the ACSCC1 cell line n=3, independent replicates (P-13, P-14, P-15)

Previously, we observed the high expression of Vimentin along with the Keratin 14 in the cells, which suggests the possibility of an increase in the partial EMT status and aggressiveness of the cells. To understand the nature of the ACSCC1 cell line, we performed the transwell invasion and migration assay in the ACSCC1 cell line. Our data revealed that ACSCC1 showed a greater invasive (Fig. 3A, B) and migratory potential (Fig. 3C, D). These data suggest that the ACSCC1 cell line showed an aggressive nature that also possesses metastatic potential. Further, we also performed the gene expression analysis of EMT markers by Real-Time PCR. We used the HEKa, as a control. Our data showed the increased expression of *Vimentin (~900 fold), Twist1 (~3.52 fold), and Zeb1 (~4.2 fold),* while a decrease in the expression of *Cdh1* *(~50% reduction)* in the ACSCC1 cell line as compared to the HEKa. We observed that increased expression of EMT markers, also enhances the invasion and migration potential. Our real-time expression data is in accord with the invasion and migration potential (Fig. 3E). Further, in vivo metastasis assay of the ACSCC1 cell line was performed by tail vein injection in the NOD/SCID mice, which showed an increased metastasis in the lungs, that was further confirmed by the H&E staining and PET-CT analysis of the lungs (Fig. 4H–J). Additionally, we performed the TEM analysis to understand the intracellular gaps between the cells. Our data showed that the ACSCC1 cell line had increased intracellular gaps between the cells (Fig. 2D, E), which can be corroborated by the invasion and migratory potential of the cells. Moreover, our TEM analysis also observed the abnormal mitochondrial morphology, suggesting impaired mitochondrial function (Fig. 2F). Importantly, we investigated the tumorigenic potential of the ACSCC1 cell line by subcutaneous injection of one million cells into the NOD/SCID mice flank. The result showed tumor formation in all three NOD/SCID mice after the 7–10 days of the injection (Fig. 4A, B). We observed the aggressive formation of the tumors by the ACSCC1 cell line, indicating the high tumorigenic potential of the cutaneous SCC cell line. Further, we performed the H&E staining in the tumor harvested from the NOD/SCID mice (Fig. 4C). The results showed a poor differentiation in the tumor derived from the xenograft. Similarly, we have also performed the H&E staining of the original patient tumor, which showed the patient tumor is also poorly differentiated (Fig. 4D). Further, we have also performed the spheroid formation assay by seeding the 5000 and 10,000 ACSCC1 cells, which showed an increased formation of spheroid capability (Fig. 4E–G). Overall, the data revealed that the cutaneous SCC cell line established by the Indian patient showed higher tumorigenic and metastatic potential.

### Determination of mutation in cancer-related genes in ACSCC1 cell line by WGS

**Fig. 5:**
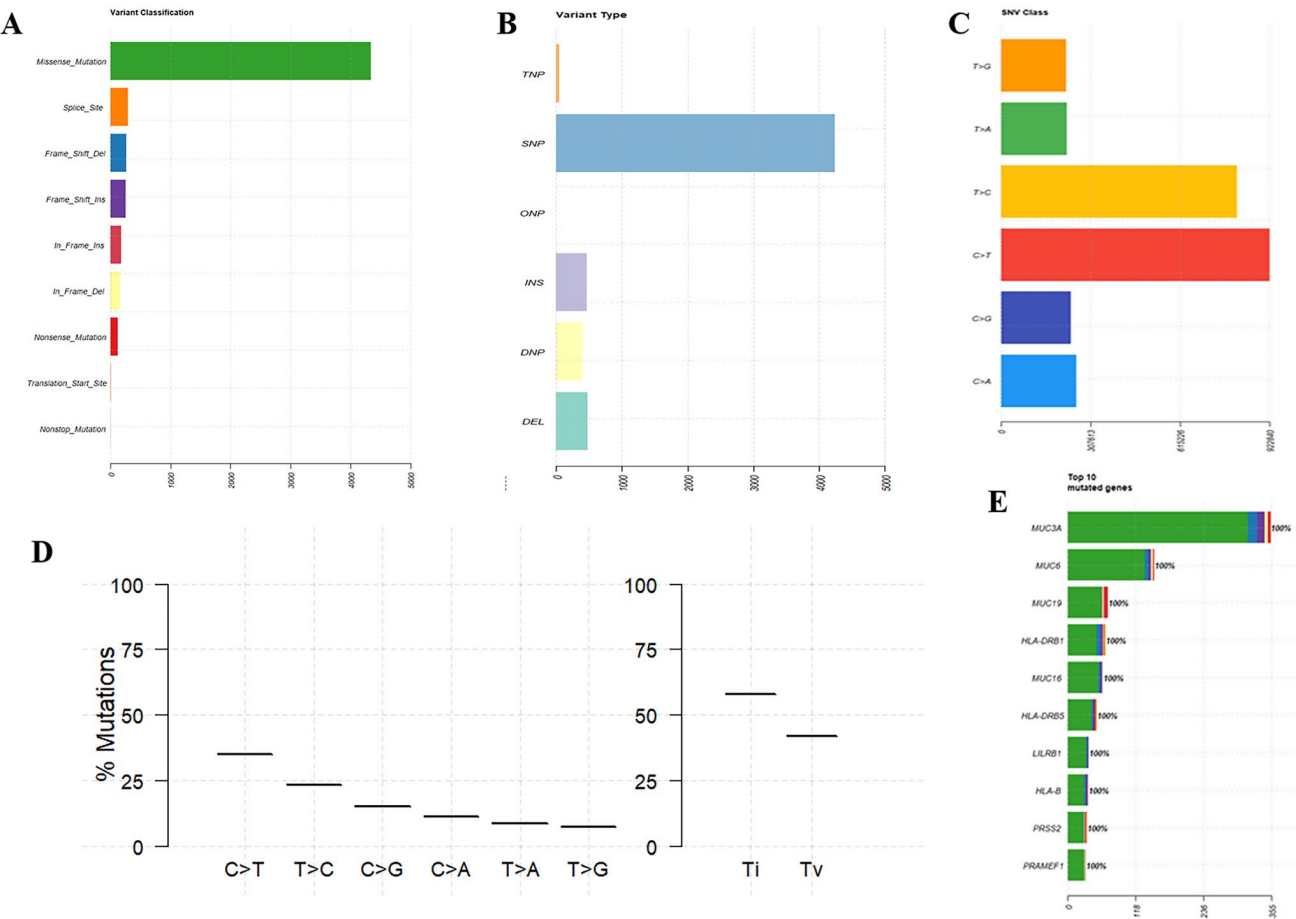
WGS analysis of ACSCC1 cell line. **A** Graphical representation of the variant classification showed a higher level of missense mutation in the ACSCC1 cell line (P-5). **B** Graphical representation of the variant type enhanced SNPs in the ACSCC1 cell line (P-5). **C** Graphical representation of single nucleotide variation in the ACSCC1 cell line (P-5). **D** Graphical representation of the percentage single nucleotide variation and Transition/Transversion in the ACSCC1 cell line (P-5) **E** Graphical representation of most mutated genes in the ACSCC1 cell line (P-5)

To understand the mutational profile of the ACSCC1 cell line, we have performed the WGS. Our data revealed that an increased level of missense mutation in the whole genome of the ACSCC1 cell line suggesting the single nucleotide substitution in the DNA, which may alter the expression of the cancer-related genes (Fig. 5A). Further, single nucleotide polymorphisms (SNPs) are most common type of genetic variation in humans, which led to increase in the development of diseases. Our data also revealed an increased number of SNPs (Fig. 5B). Moreover, we found ~35% of C>T transition in the entire genome of the ACSCC1 cell line (Fig. 5C, D), which is frequently observed in the different types of cancer. Our data showed that the MUC3A gene is highly mutated, which consists of missense mutation, frameshift insertion, frameshift deletion, and nonsense mutation. Additionally, we also observed the missense mutation in genes such as CDKN2A, TP53, NOTCH1, MYC, RB1, APC, CCND1, PIK3CA, TP63 and PDGFRA associated with different SCC (Supp. Table 4). Overall, our WGS data showed that mutations associated with cutaneous SCC genes enhance the tumorigenic and metastatic potential of the ACSCC1 cell line.

## Discussion

India perceives a small percentage of diagnosed cancers attributed to cutaneous malignancies. Across the globe, basal squamous cell carcinoma (BCC) is the most prevalent form of skin cancer. However, 30–60% of cutaneous SCC metastasized and showed poor overall survival [30]. Advanced-stage cutaneous SCC presents a complex clinical scenario characterized by resistance to conventional surgical and radiotherapeutic modalities, depicting an uncommon subgroup within the domain of cutaneous SCC [31, 32]. Currently, there are very few well-characterized cutaneous SCC cell lines (A431, SCC12, SCC13B, UT-SCC-12 A, UT-SCC-91, UT-SCC-105, UT-SCC-111, UT-SCC-118, and HCB-541) [13, 15, 17, 19, 33]. However, there are no reports on cutaneous SCC cell lines derived from Indian patients. Developing such cell lines is crucial for understanding tumor heterogeneity and potential molecular variations between Indian and Western populations. The epidemiology of cutaneous SCC shows significant diversity across the Western and Southeast Asian populations, likely due to genetic differences, highlighting the need for region-specific models in research [34–36]. Therefore, the cell line derived from the advanced-stage cutaneous SCC Indian patient may be helpful in developing therapeutic strategies for the patients and molecular validation of the differentially expressed genes.

In our study, the cell line was derived directly from the primary tumor of a treatment-naive, advanced-stage cutaneous SCC patient, thereby avoiding any genotypic or phenotypic alterations associated with patient-derived xenograft generation. This cell line exhibits anchorage-independent growth without the need for feeder cells. The morphology and clustered growth patterns affirm the epithelial characteristics of the cell line. Additionally, the presence of Keratin 8 and Keratin 14 expression further strengthens the epithelial identity, given that keratins are specific to epithelial cells [37]. STR profiling was done on both the ACSCC1 cell line and in original tumor that matched perfectly. Further, this STR profile was analyzed with the known STR profiles of the cell lines in the DSMZ data base that did not show any match suggesting that the ACSCC1 is a novel cell line[38]. The ACSCC1 cell line did not significantly match the existing database. Additionally, the cell line exhibited a difference in morphology, expression of keratins, and STR profiles, which confirms the uniqueness of the cell line. HPV is one of the risk factors associated with cutaneous SCC [39]. We evaluated the HPV expression by the nested PCR using the My09/My11 and Gp5+/Gp6+ primers encoded for the capsid of a virus. Our data showed that the ACSCC1 cell line is free from HPV infection. Further, analysis of the DNA ploidy in cancer cells offers insights into the disease’s aggressiveness, metastatic capabilities, and prognosis [40, 41]. The DNA content of the ACSCC1 cell line was higher than the diploid lymphocytes. The ACSCC1 cell line showed hyperdiploid DNA content with a DNA index of 1.88, suggesting the rapid growth of the cancer cells. A study has proved that the rapid growth of cancer cells results in aberrant karyokinesis with an increased ploidy level [42]. Furthermore, the karyotyping analysis of the ACSCC1 cell line showed the hyperdiploid karyotype. ACSCC1 showed chromosome heterogeneity, often observed in solid tumors [43]. The hyperdiploid karyotype has been observed in well-established primary cell cultures such as buccal mucosa (head and neck), breast cancer, and cutaneous SCC [19, 20, 44].

In vivo, tumorigenesis assay determines the tumorigenic potential of the established cell line. The ACSCC1 cell line showed a higher tumorigenic potential, which showed that the cell line could be used to establish the xenograft models and test the efficacy of the therapeutic reagents. Importantly, the majority of deaths from cancer happen due to metastasis [45]. We evaluated the invasive and migratory potential of the ACSCC1 cell line by the trans-well assay. ACSCC1 showed a higher invasion and migration of the cells. Further, we have also confirmed the metastatic potential of the ACSCC1 cell line by in vivo metastasis assay in the NOD/SCID mice, which showed an increased lung metastasis in the NOD/SCID mice. Moreover, we observed the similarities between invasion and migration by performing the investigated ultrastructure analysis of the ACSCC1 cells by TEM. We showed the increased intracellular gaps between the ACSCC1 cells. These results agree with the invasion and migration data because higher intracellular gaps correspond to increased invasion and migration. The primary oral cancer cell line ACOSC4 showed increased intracellular gaps and invasion and migration potential [22]. Further, we checked the EMT status of the cell line by Real-Time PCR because EMT is one factor contributing to metastasis. Our study found increased Vimentin, Twist1, and Zeb1 expression in the ACSCC1 cell line. Earlier reports revealed that an increased expression of Vimentin, Zeb1, and Twist1 enhances migratory properties in the OSCC [46–48]. Further, our WGS data showed a plethora of missense mutations in the genome of the ACSCC1 cell line. Several studies have reported that missense mutations are essential and play a vital role in carcinogenesis [49]. Moreover, our data represented an increased number C>T mutations in the ACSCC1 cell line. Previously, the TCGA database of 450 cases of cutaneous melanoma showed majority of missense mutations, and most of them have C>T transitions [50]. Importantly, we found missense mutations in the several genes such as TP53, CDK2NA1, NOTCH1, TP63 in the ACSCC1 cell line. It has been reported that missense mutations in the CDK2NA1 and TP53 genes are associated with the HNSCC and cutaneous SCC [19, 51]. Further, our data showed that the MUC3A gene is highly mutated in the entire genome of the ACSCC1 cell line. MUC3A mutation is associated with poor overall survival in chronic myeloid leukemia [52] and overexpression of MUC3A enhances the metastasis, which showed poor prognosis in gastric cancer and non-small cell lung cancer [53, 54].

To our knowledge for the first time, we report the establishment of the novel cutaneous SCC cell line ACSCC1 from the advanced stage treatment-naïve Indian patient that showed higher tumorigenic, invasive, and metastatic potential. This cell line can be used to understand the molecular drivers of tumor growth and to study the role of tumor microenvironment interactions and their impact on tumor progression. Moreover, the ACSCC1 cell line can be used in pre-clinical orthotopic models that would provide a platform for studying drug resistance mechanisms in cutaneous SCC. It can be useful for the identification of molecular markers and signaling pathways that are involved in the invasion and metastasis specific to skin SCC. Further, the aberrant mitochondrial morphology observed in ACSCC1 suggests alterations in mitochondrial dynamics and potentially altered energy metabolism. This offers an opportunity to investigate the mechanism that may selectively target cancer cells with altered bioenergetics.

## Supplementary Information

Below is the link to the electronic supplementary material.

Supplementary file1 (DOCX 21 KB)

## Data Availability

The datasets generated and/or analyzed during the current study are available from the corresponding author upon reasonable request.
