# Supplementary material for: Establishment and molecular characterization of the novel cutaneous squamous cell carcinoma cell line from advanced-stage Indian patient
Source: Hum Cell. 2025 May 25;38(4):108. doi: 10.1007/s13577-025-01237-4 (PMC12104104; doi:10.1007/s13577-025-01237-4)
Supplement: Supplementary file 1 — Supplementary file1 (DOCX 21 KB) [file 13577_2025_1237_MOESM1_ESM.docx]

**Supplementary Tables:**

| **Primer** | **Oligonucleotide sequence in 5’ to 3’ orientation** |
| --- | --- |
| GAPDH | Forward: GAAGGTCGGAGTCAACGGATTT  Reverse: GATGACAAGCTTCCCGTTCTCA |
| Vimentin | Forward: AGTCCACTGAGTACCCGGAGAC  Reverse: CATTTCACGCATCGGCGTTC |
| Twist 1 | Forward: CGGGAGTCCGCAGTCTTA  Reverse: GCTTGAGGGTCTGAATCTTG |
| Zeb 1 | Forward: TTACACCTTTGCATACAGAACCC  Reverse: TTTACGATTACACCCAGACTGC |
| E- cadherin | Forward: CGAGAGCTACACGTTCACGG  Reverse: GGGTGTCGAGGGAAAAATAGG |
| My09 | Forward: CGTCCAAGAGGATACTGATC |
| My11 | Reverse: GCCCAGGGTCTATAACAATGG |
| Gp5+ | Forward: TTTGTTACTGTGGTAGATACTAC |
| Gp6+ | Reverse: GAAAAATAAACTGTAAATCATATTC |

**Supplementary Table 1:** List of Real-time PCR primers

| **Immunofluorescence Assay (IFA)** | | |
| --- | --- | --- |
| Vimentin | 1:200 | Abcam |
| E-cadherin | 1:150 | Abcam |
| Keratin-8 | 1:200 | Novus biologicals |
| Keratin-14 | 1:200 | Abcam |
| Anti-rabbit | 1:200 | Jackson Laboratories |
| Anti-mouse | 1:200 | Jackson Laboratories |

**Supplementary Table 2:** List of Antibodies

| **Markers** | **ACSCC1** | | **Original Patient Tumor** | |
| --- | --- | --- | --- | --- |
| **TH01** | **7** | **8** | **7** | **8** |
| **D5S818** | **13** |  | **13** |  |
| **D13S317** | **8** | **10** | **8** | **10** |
| **D7S820** | **12** |  | **12** |  |
| **D16S539** | **11** | **14** | **11** | **14** |
| **CSF1PO** | **10** |  | **10** |  |
| **vWA** | **16** |  | **16** |  |
| **TPOX** | **10** | **11** | **10** | **11** |
| **Amelogein** | **X** | **Y** | **X** | **Y** |

**Supplementary Table 3:** STR profile of ACSCC1 cell line and original patient Tumor

| **Gene name** | **Variant classification** | **Variant Type** | **Nucleotide Variation** | **Exon** | **Protein** |
| --- | --- | --- | --- | --- | --- |
| CDKN2A | Missense Mutation | SNP | c.442G>A | 02/03 | p.Ala148Thr |
| MYC | In Frame Deletion | DEL | c.154_156del | 02/03 | p. Gln52del |
| NOTCH1 | Frame Shift Deletion | DEL | c.4116_4119del | 25/34 | p. Leu1373AlafsTer71 |
| TP53 | Missense Mutation | SNP | c.404G>T | 05/11 | p. Cys135Phe |
| PIK3CA | Missense Mutation | SNP | c.1633G>A | 10/21 | p. Glu545Lys |
| PDGFRB | Missense Mutation | SNP | c.1972C>A | 14/23 | p. Leu658Ile |
| ARID1A | Missense Mutation | SNP | c.3964A>G | 16/20 | p. Ser1322Gly |
| APC | Missense Mutation | SNP | c.2190G>T | 16/16 | p. Met730Ile |
| RB1 | Missense Mutation | SNP | c.296T>C | 02/02 | p. Leu99Ser |
| CCND1 | In Frame Deletion | DEL | c.839_841del | 05/05 | p. Glu280del |
| MMP8 | Missense Mutation | SNP | c.1348C>T | 10/10 | p. Gln450Ter |
| TP63 | Missense Mutation | SNP | c.2025C>G | 14/14 | p. Ile675Met |
| KIT | Missense Mutation | SNP | c.530G>A | 03/21 | p. Arg177His |

**SNP= Single Nucleotide Polymorphism DEL= Deletion**

**Supplementary Table 4: Mutation profile of ACSCC1 cell line**
